# Hydatid Cyst Diagnosed by Endoscopic Ultrasound

**DOI:** 10.1155/2016/7260392

**Published:** 2016-04-20

**Authors:** F. Castro-Poças, Tarcísio Araújo, André Coelho, Donzilia Silva, Isabel Pedroto

**Affiliations:** ^1^Department of Ultrasound and Department of Gastroenterology, Santo António Hospital, Porto Hospital Center, Porto, Portugal; ^2^Institute of Biomedical Sciences Abel Salazar, University of Porto, Porto, Portugal; ^3^Department of Pathology, Santo António Hospital, Porto Hospital Center, Porto, Portugal; ^4^Department of Surgery, Santo António Hospital, Porto Hospital Center, Porto, Portugal

## Abstract

A 69-year-old female with unremarkable past history underwent endoscopy for dyspepsia. She denied weight loss or anorexia. Upper endoscopy revealed a bulge in the lesser curvature and posterior wall of the stomach with 4-5 cm. Endoscopic ultrasound was performed which showed a heterogeneous lesion, anechogenic in the major part, with a floating membrane inside, the greatest diameter of 90.8 × 17.2 mm, originated in the left liver lobe. Surgical resection was performed. Pathologic examination revealed a cystic lesion with an acellular thick fibrous wall, surrounded by a conspicuous inflammatory reaction. The cyst wall revealed a characteristic lamellar pattern of the fibers. In the internal surface of the lesion, there were remains of membranous structures, amidst which a vestigial Protoscolex was noted. In the presented case, a floating membrane was observed, which is a pathognomonic feature, establishing the diagnosis of hydatid cyst type 3. Fine needle aspiration guided by ultrasound was not performed due to the certainty in the diagnosis. To the authors' knowledge, these are the first images by endoscopic ultrasound of hydatid cyst of liver presented as a bulge in the stomach with pathognomonic features, which allowed the definitive diagnosis with no need for further diagnostic tests.

## 1. Case Presentation

**Figure 1:**
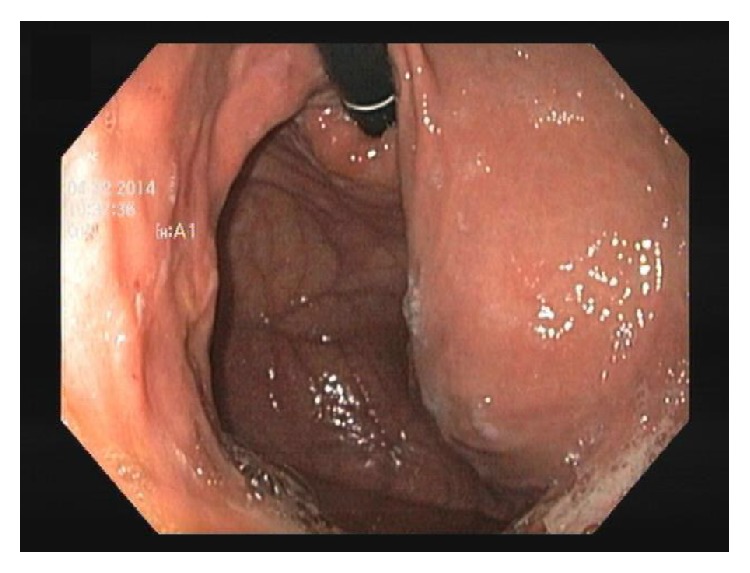
Bulge in the lesser curvature and posterior wall of the stomach.

**Figure 2:**
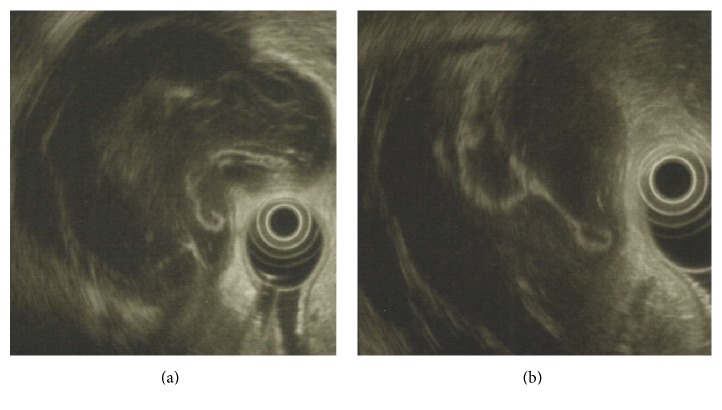
Heterogeneous lesion with a floating membrane inside.

A 69-year-old female with unremarkable past history underwent an upper endoscopy for dyspepsia. She denied weight loss, anorexia, asthenia, or vomits. Physical examination was normal. Upper endoscopy revealed a bulge in the lesser curvature and the posterior wall of the stomach with 4-5 cm (Figure 1). Endoscopic ultrasound (EUS) was performed and showed a heterogeneous lesion, mainly anechogenic one, with a floating membrane inside, and greatest diameter of 90.8 × 17.2 mm, originated in the liver left lobe (Figure 2). The diagnosis of a hydatid cyst type 3 was made. A surgical resection was performed. The patient recovered without complications.

**Figure 3:**
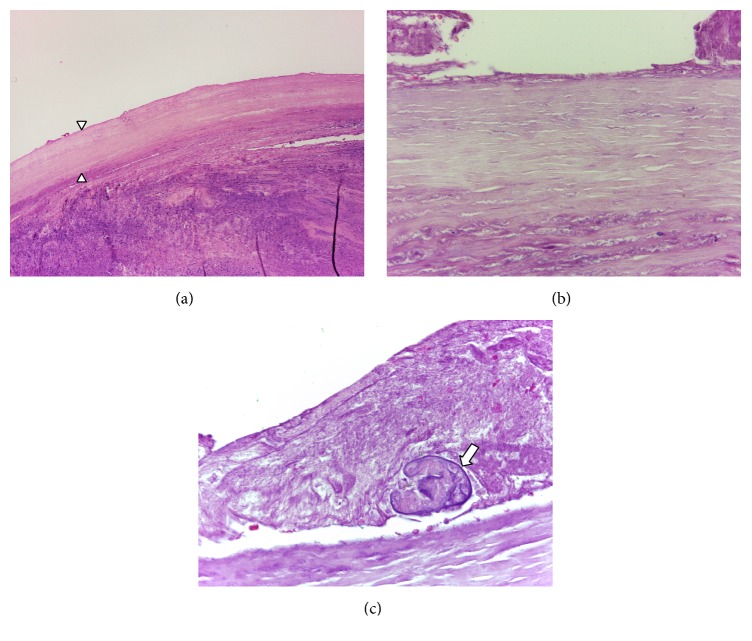
Pathologic examination: (a) acellular thick fibrous wall (arrowheads) (H&E stain, ×40); (b) the cyst wall revealing a characteristic lamellar pattern of the fibers (H&E stain, ×400); and (c) vestigial Protoscolex amidst membranous structures (arrow) (H&E stain, ×400).

Pathologic examination (Figure 3) revealed a cystic lesion with acellular thick fibrous wall, surrounded by a conspicuous inflammatory reaction. The cyst wall revealed a characteristic lamellar pattern of the fibers. In the internal surface of the lesion, there were remains of membranous structures amidst, in which a vestigial Protoscolex was noted.

## 2. Discussion

Hydatid disease is a worldwide parasitic infestation by tapeworms of* Echinococcus* type and represents a substantial disease burden [1]. World Health Organization has established a classification based on ultrasound features, divided in 5 types: 1, 2, 3, 4, and 5 [2].

In English literature, we found only one report in which the EUS contributed decisively to the diagnosis of a liver hydatid cyst, allowing for sample collection with fine needle aspiration in a lesion without imagiologic diagnosis [3].

In the presented case, a floating membrane was observed, which is a pathognomonic feature, establishing the diagnosis of hydatid cyst type 3. Fine needle aspiration guided by ultrasound was not performed due to the certainty in the diagnosis.

To the author's knowledge, these are the first images by EUS of a hydatid cyst of liver presented as a bulge in the stomach with pathognomonic features, which allowed for the definitive diagnosis with no need for further diagnostic tests.
